# RUNX1 Plays an Important Role in Mediating BMP9-Induced Osteogenic Differentiation of Mesenchymal Stem Cells Line C3H10T1/2, Murine Multi-Lineage Cells Lines C2C12 and MEFs

**DOI:** 10.3390/ijms18071348

**Published:** 2017-06-23

**Authors:** Caixia Ji, Xiaohua Liu, Li Xu, Tingting Yu, Chaoqun Dong, Jinyong Luo

**Affiliations:** Department of Laboratory Medicine, M.O.E. Key Laboratory of Laboratory Medicine Diagnostics, Chongqing Medical University, Chongqing 400016, China; 2014111003@stu.cqmu.edu.cn (C.J.); 2014111004@stu.cqmu.edu.cn (X.L.); 2015111028@stu.cqmu.edu.cn (L.X.); 2016110976@stu.cqmu.edu.cn (T.Y.); 2015110783@stu.cqmu.edu.cn (C.D.)

**Keywords:** RUNX1, BMP9, MSCs, osteogenic differentiation

## Abstract

As one of the least studied bone morphogenetic proteins (BMPs), BMP9 is highly capable of promoting osteogenic differentiation. However, the underlying mechanism involved remains largely unknown. Recent studies have demonstrated that RUNX1 (runt-related transcription factor 1) is essential in osteoblast/chondrocyte maturation. In this study, we investigated the function of RUNX1 in BMP9-induced osteogenic of murine mesenchymal stem cell line (C3H10T1/2) and murine multi-lineage cell lines (C2C12 and MEFs). Our data showed that BMP9 promoted the endogenous expression of RUNX1 in C3H10T1/2, C2C12 and MEFs. Moreover, RUNX1 was probably a direct target of BMP9/Smad signaling. BMP9-induced osteogenic differentiation was enhanced by overexpression of RUNX1, whereas inhibited by knockdown RUNX1 in C3H10T1/2, C2C12 and MEFs. Further mechanism studies demonstrated that RUNX1 might affect BMP9-induced phosphorylation of Smad1/5/8, but not the phosphorylation of p38 and ERK1/2.Our results suggest that RUNX1 may be an essential modulator in BMP9- induced osteogenic differentiation of MSCs (Mesenchymal stem cells).

## 1. Introduction

Bone defect, as a refractory disease, could lead to disabling pain and functional limitation [1,2]. Bone tissue engineering and regenerative medicine based on stem cells combined with tissue-engineered scaffolds and cytokines have shown a promising potential in regenerating bone defects [3,4,5]. MSCs are non-hematopoietic, adult multipotent stem cells that can be isolated from multiple sources (such as trabecular bone, periosteum, synovium, adipose tissue, skeletal muscle, deciduous teeth and peripheral blood). When stimulated with lineage-specific induction media, MSCs differentiate toward osteoblastic, chondrogenic, myogenic, and adipogenic phenotypes [6,7,8]. With widespread source and characteristic of multipotent differentiation, MSCs have been widely used in bone tissue engineering in clinical setting [9,10,11]. Bone morphogenetic proteins (BMPs), which are members of the transforming growth factor-β (TGF-β) superfamily, are now acknowledged to be involved in regulating embryonic development and differentiation as well as cellular function [12,13]. Besides, BMPs have been shown to induce the osteogenic differentiation of murine multi-lineage cells (MMCs) and promote bone formation during bone remodeling [14]. To date, more than 20 BMPs have been identified, among these, BMP2, BMP6 and BMP7 are known to play an important role in the process of MSCs towards osteogenic differentiation [15]. Additionally, BMP2 and BMP7 have been used in clinical applications [16,17]. However, it remains unclear whether BMP2 and BMP7 are in fact the most potent BMPs in inducing osteogenic differentiation and bone formation [18,19]. Therefore, we have conducted a comprehensive analysis of the osteogenic activity of 14 human BMPs and demonstrated that bone morphogenetic protein 9 (BMP9), is more potent in promoting the osteogenic differentiation of MSCs, both in vitro and in vivo [20]. Furthermore, a set of pivotal transcription factors such as Id1 (inhibitor of differentiation 1), Id2 (inhibitor of differentiation 2), Id3 (inhibitor of differentiation 3), DLX5 (Distal-less homeobox 5), RUNX2 (runt-related transcription factor 2) and OSX (Osterix) play an important role in regulating BMP9-induced osteogenic differentiation of MSCs [21,22,23]. It has been reported that a variety of signaling pathways such as canonical BMPs/Smad and non-canonical mitogen-activated protein kinase (MAPKs) signaling pathway are involved in BMP9-induced osteogenic differentiation of MSCs [24]. Additionally, fibroblast growth factor-2 (FGF-2), epidermal growth factor (EGF), BMP type I receptor, ALK1 (Extracellular signal-regulated protein kinase 1), ALK2 (Extracellular signal-regulated protein kinase 2) and microRNAs are important regulators in BMP9-induced osteogenesis [25,26,27,28]. Despite these valuable findings, BMP9 remains the least studied BMPs, and the molecular mechanism underlying BMP9-induced osteogenic differentiation is still unclear and warrant further investigation.

The runt-related (RUNX) family of transcription factors, which bind DNA as heterodimers with CBFβ, are known to play critical roles in embryonic development. To date, three members (RUNX1, RUNX2 and RUNX3) have been characterized in RUNX family. Generally, RUNX2 is necessary for bone formation, and RUNX3 for gastrointestinal and nervous system development [29]. RUNX1 is a key transcription factor that regulates hematopoietic stem cells and hematopoiesis [29], however, there is growing evidence that RUNX1 participates in various maturational processes required for skeletal developmental events [30,31]. Overexpression of RUNX1 in MSCs has been shown to promote chondrocyte development [32]. RUNX1 deletion by the Prrx1 (paired-related homeobox transcription factor-1)-Cre mouse line causes a defect formation of mineralization which affects the formation of the sternum [33]. We have demonstrated that RUNX2 is involved in BMP9-induced osteogenic differentiation of MSCs [34]. As RUNX1 shares similar structure with RUNX2 and co-expressed in many skeletal elements [29], we hypothesized that RUNX1 may be involved in BMP9-induced osteogenic differentiation. In this study, we investigated the effect of RUNX1 on BMP9-induced osteogenic differentiation in C3H10T1/2, C2C12 and MEFs cell lines. We found that BMP9 increased RUNX1 expression in these cell lines. Chromation immunoprecipitation (ChIP) analysis indicated that RUNX1 might be a direct target of the BMP/Smad signaling. BMP9-induced osteogenic differentiation was enhanced by overexpression of RUNX1, whereas was inhibited by knockdown RUNX1. Mechanically, we confirmed that RUNX1 might affect BMP9-induced phosphorylation of Smad1/5/8. These findings suggest that RUNX1is probably to regulate BMP9-induced osteogenic differentiation through canonical Smad signaling.

## 2. Results

### 2.1. RUNX1 Is Upregulated by BMP9 in MSCs and MMCs

**Figure 1:**
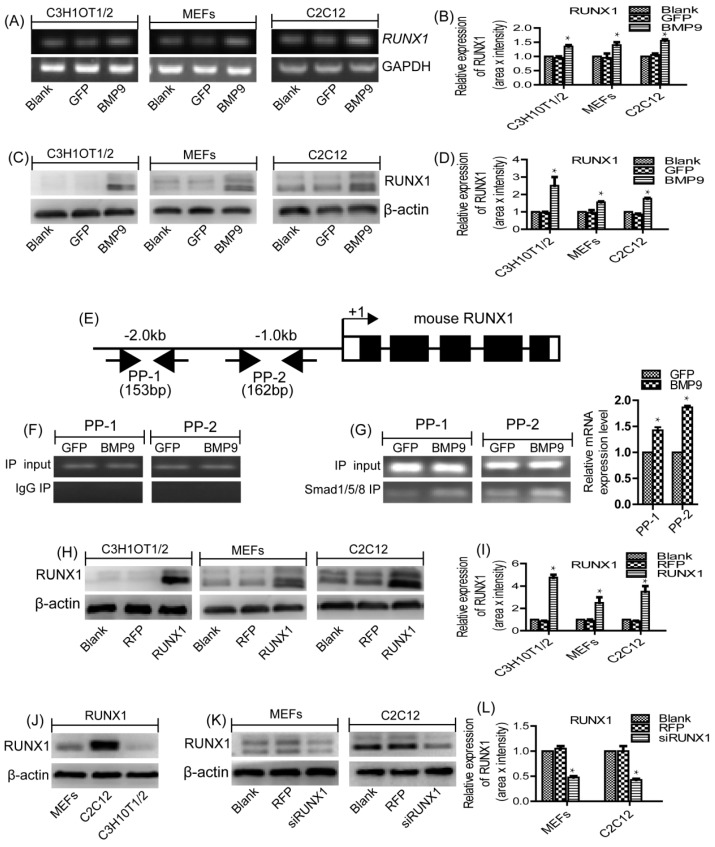
BMP9 enhanced the expression level of RUNX1 in MSCs and MMCs, RUNX1 was a direct target of BMP9/Smad signaling. (**A**,**B**) The gene expression level of RUNX1 was detected by RT-PCR in C3H10T1/2, C2C12 and MEFs after Ad-BMP9 infection for 48 h. (**C**,**D**) The protein expression level of RUNX1 was detected by Western blotin C3H10T1/2, C2C12 and MEFs after Ad-BMP9 infection for 72 h. (**E**) A schematic presentation of the 2.0 kb promoter region of mouse RUNX1. (**F**,**G**) ChIP analysis of the mouse RUNX1 promoter. MEFs cells were infected with Ad-GFP or Ad-BMP9. Cells were cross-linked. The cross-linked cells were lysed and subjected to sonication, following immunoprecipitation with Smad1/5/8 antibody or IgG. The retrieved genomic DNA was amplified by RT-RCR using the two pairs of primers, PP-1 and PP-2. The genomic DNA input was comparable among the samples. (**H**,**I**) the protein expression level of RUNX1 was detected by Western blot in C3H10T1/2, C2C12 and MEFs after Ad-RUNX1 infection for 72 h. (**J**) The protein expression level of RUNX1 was detected by Western blot in C3H10T1/2, C2C12 and MEFs. (**K**,**L**) The protein expression level of RUNX1 was detected by Western blot in C2C12 and MEFs after Ad-siRUNX1 infection for 72 h. GAPDH and β-actin were used as loading control, separately. * *p* < 0.05, as compared with control group. RUNX: runt-related transcription factor; MSCs: Mesenchymal stem cells; MMCs: murine multi-lineage cells; RT-PCR: Semi-Quantitative PCR; ChIP: Chromatin Immunoprecipitation; Ad-GFP: Adenovirus carrying green fluorescent protein gene; Ad-BMP9: :Adenovirus carrying bone morphogenetic protein 9 gene; IgG: Immunoglobulin G; GAPDH: Glyceraldehyde 3-phosphate-dehydrogenase.

Previous study has proved that BMP9 is one of the most osteogenic BMPs [20]. Through gene expression profiling analysis, we have found several downstream targets that may play important roles in mediating BMP9-induced osteogenic differentiation [35,36,37]. In this current study, we explored the role of RUNX1 in BMP9-induced osteogenic differentiation of MSCs and MMCs. Firstly, we sought to determine if BMP9 can affect the expression of endogenous RUNX1. We found that BMP9 can effectively increase the mRNA level (Figure 1A,B) and protein level (Figure 1C,D) of RUNX1 in C3H10T1/2, C2C12 and MEFs.

### 2.2. RUNX1 Is a Direct Target of BMP9/Smad Signaling Pathway

We next tested if RUNX1 is a direct target of BMP9 Smad signaling by analyzing if RUNX1 promoter can interact with the BMP-specific Smad1/5/8. We conducted ChIP analysis using Smad1/5/8 antibody or isotype IgG to pull down genomic DNA. Two pairs of PCR primers (PP-1 and PP-2) were designed to analyzed the possible presence of BMP R-Smad binding site(s) in the mouse 2.0-kb RUNX1 promoter region (Figure 1E). For ChIP analysis, MEFs were infected with Ad-GFP or Ad-BMP9 and cross-linked genomic DNA protein complexes were immunoprecipitated with anti-Smad1/5/8 antibody. Then, the retrieved genomic DNA was amplified by RT-PCR using the two pairs of primers, PP-1 and PP-2. While the genomic DNA input was comparable among the samples, both PP-1 and PP-2 was shown to amplify more expected product in BMP9-stimulated cells, although the basal level amplification was readily detectable (Figure 1F,G). The above result suggests that RUNX1 may be regulated by BMP9 through BMP9-specific Smad1/5/8. To confirm the effect of RUNX1 on BMP9 osteoinductive activity, we constructed recombinant adenovirus expressing RUNX1 (Ad-RUNX1), and confirmed the robust transgene expression of Ad-RUNX1 in MSCs and MMCs (Figure 1H–I). Subsequently, we examined the endogenous expression levels of RUNX1 in different cell lines and demonstrated RUNX1 was highly expressed in C2C12 and MEFs cells as compared to C3H10T1/2 cells (Figure 1J). Therefore, we constructed recombinant adenovirus expressing small interference RNA (siRNA) for RUNX1 (Ad-siRUNX1) and validated the knockdown efficiency of Ad-siRUNX1 in two cell lines (C2C12 and MEFs) (Figure 1K–L).

### 2.3. Effect of RUNX1 on BMP9-Induced Early Osteogenic Marker-Alkaline Phosphatase (ALP)

**Figure 2:**
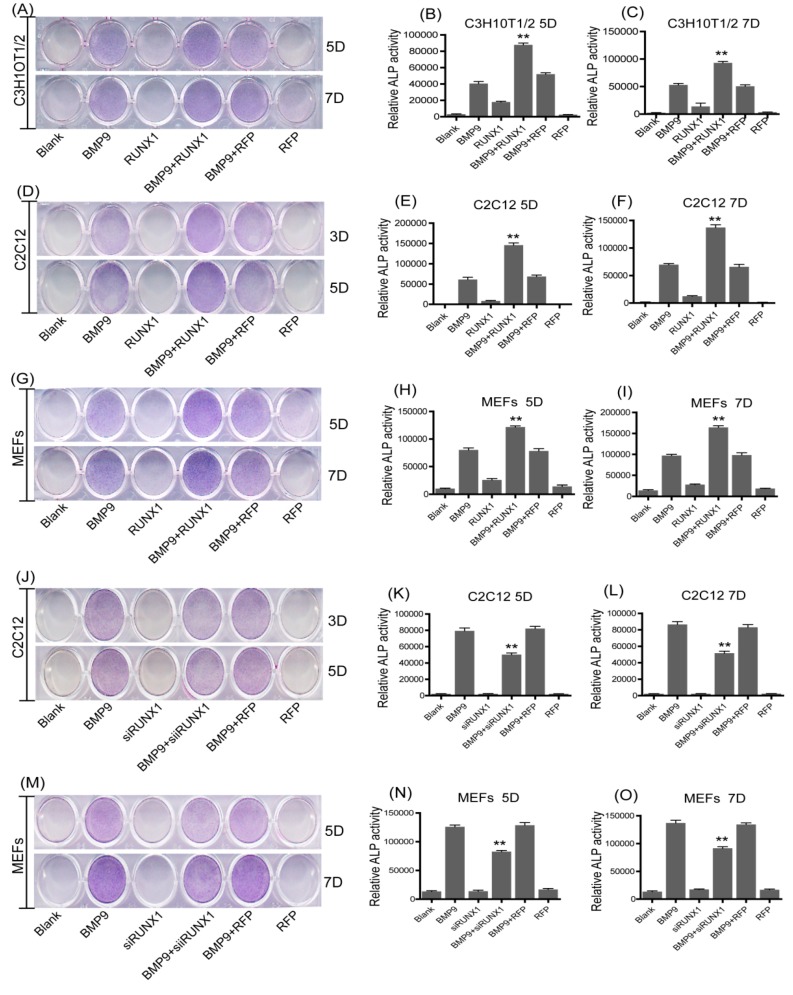
Effect of RUNX1 on the bone morphogenetic protein 9 (BMP9)-induced early osteogenic differentiation of MSCs and MMCs. Cells were infected with Ad-RUNX1 or Ad-siRUNX1, followed by treatment with optimal BMP9-CM. ALP activity was detected by histochemical staining assay and chemiluminescence assay at five days(5D)and seven days(7D).Overexpression of RUNX1 promoted BMP9-induced ALP activity in: C3H10T1/2 (**A**–**C**); C2C12 (**D**–**F**); and MEFs (**G**–**I**). Inhibition of RUNX1 decreased BMP9-induced ALP activity in: C2C12 (**J**–**L**); and MEFs (**M**–**O**). ** *p* < 0.01, as compared with BMP9 group.

We have found that BMP9 can increase the RUNX1 expression in MSCs and MMCs. This finding prompted us to evaluate the effect of RUNX1 on BMP9-induced osteogenic differentiation. Therefore, C3H10T1/2, C2C12 and MEFs were infected with Ad-RFP or Ad-RUNX1, followed by treated with BMP9 Conditional Medium (BMP9-CM), ALP staining assays and ALP activity assay were used to determine the early differentiation of these cell lines. We found that BMP9-induced ALP activity was increased by Ad-RUNX1 in C3H10T1/2 (Figure 2A–C), C2C12 (Figure 2D–F) and MEFs (Figure 2G–I).To complement these results, we also infected C2C12 and MEFs with small interference RNA for RUNX1 (Ad-siRUNX1). Conversely, BMP9-induced ALP activity was significantly decreased upon Ad-siRUNX1 treatment in C2C12 and MEFs (Figure 2J–O). Collectively, these results suggest that RUNX1 can regulate BMP9-induced early osteogenenic differentiation of MSCs and MMCs.

### 2.4. Effect of RUNX1 on BMP9-Induced Late Osteogenic Differentiation

**Figure 3:**
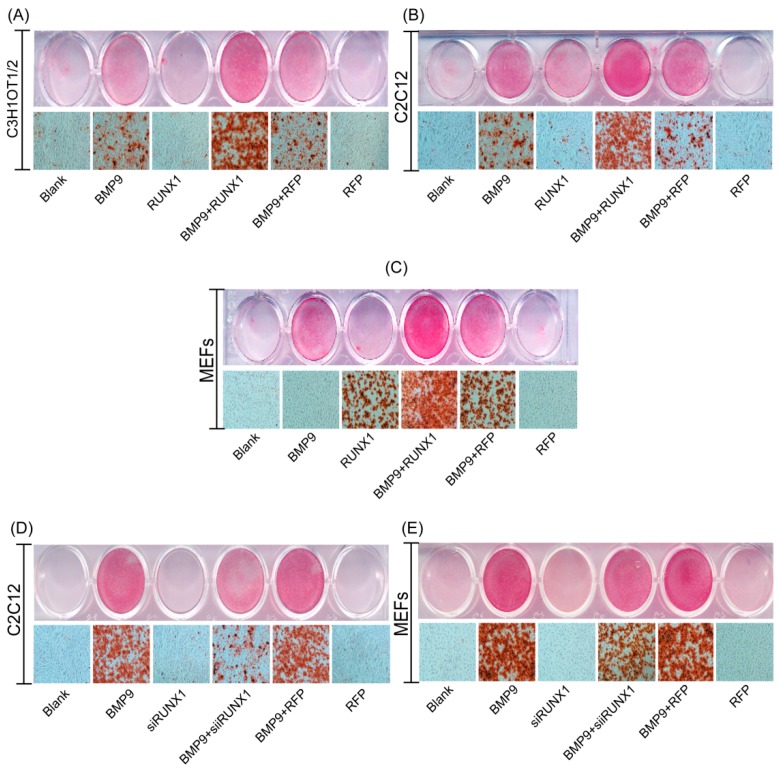
Effect of RUNX1 on BMP9-induced late osteogenic differentiation of MSCs and MMCs. Cells were infected with Ad-RUNX1or Ad-siRUNX1, followed by treatment with optimal BMP9-CM. Matrix mineralization was assessed at 14 days post-treatment by Alizarin Red S staining. Overexpression of RUNX1 promoted BMP9-induced calcium deposition in: C3H10T1/2(**A**); C2C12 (**B**); and MEFs (**C**). Inhibition of RUNX1 decreased BMP9-induced calcium deposition in: C2C12 (**D**); and MEFs (**E**). Magnification, ×100.

ALP is an early osteogenic marker, and it cannot evaluate late osteogenic stage accurately. Thus, we thought to test the effect of RUNX1 on BMP9-induced late osteogenic marker matrix mineralization. We found that Ad-RUNX1 promoted BMP9-induced matrix mineralization in C3H10T1/2 (Figure 3A), C2C12 (Figure 3B) and MEFs (Figure 3C). On the contrary, Ad-siRUNX1 decreased the BMP9-induced matrix mineralization in C2C12 (Figure 3D) and MEFs (Figure 3E). These results strongly suggest that RUNX1 can affect BMP9-induced late osteogenic differentiation of MSCs and MMCs.

### 2.5. Effect of RUNX1 on BMP9-Induced mRNA Expression Levels of Pivotal Osteogenic Markers

**Figure 4:**
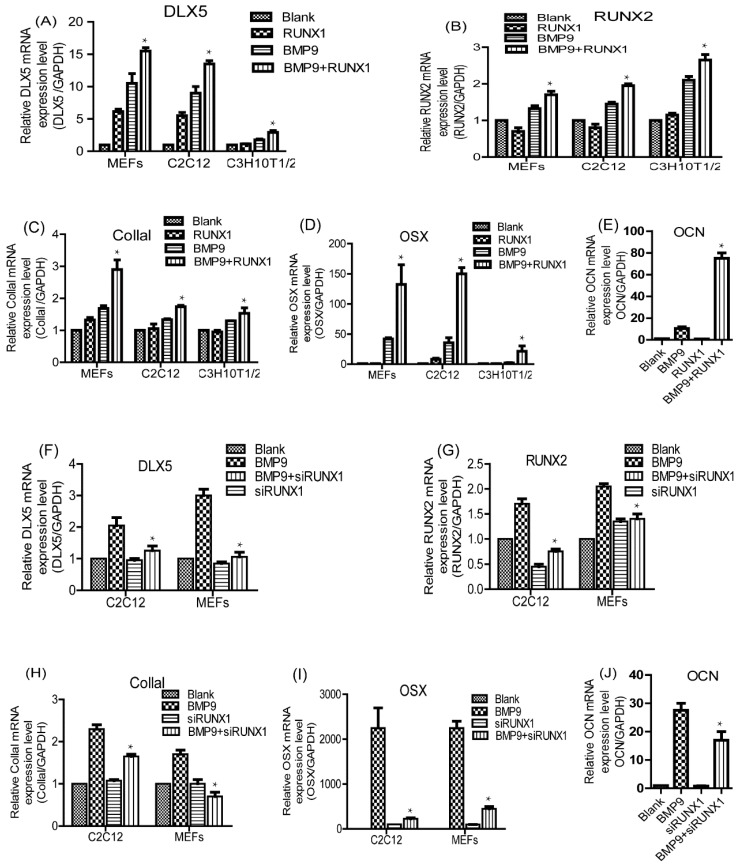
Effect of RUNX1 on BMP9-induced mRNA expression levels of pivotal osteogenic markers. Cells were infected with Ad-RUNX1 or Ad-siRUNX1, and then treated by optimal BMP9-CM.Total RNA was extracted from the cultures and was measured by RT-qPCR. Overexpression of RUNX1 promoted BMP9-induced mRNA levels of DLX5 (Distal-less homeobox 5) (**A**); RUNX2 (**B**); CollaI (**C**); and OSX (**D**), in C3H10T1/2, C2C12 and MEFs. (**E**) Overexpression of RUNX1 promoted BMP9-induced mRNA levels of OCN in MEFs. Inhibition of RUNX1 decreased BMP9-induced mRNA levels of: DLX5 (**F**); RUNX2 (**G**); CollaI (**H**); and OSX (**I**) in C2C12 and MEFs. (**J**) Inhibition of RUNX1 decreased BMP9-induced mRNA levels of OCN in MEFs. * *p* < 0.05, as compared with BMP9 group.

Previous studies have demonstrated that OSX, CollaI (Collagen 1), DLX5, RUNX2 and OCN (osteocalcin) are pivotal osteogenic markers in BMP9-induced osteogenic differentiation of MSCs [14,25]. Next, we considered evaluating the effect of RUNX1 on BMP9-induced pivotal osteogenic markers by using RT-qPCR (quantitative real-time PCR) analysis. We found that BMP9-induced gene expression of OSX, CollaI, DLX5, RUNX2 and OCN was strongly increased by Ad-RUNX1 in C3H10T1/2, C2C12 and MEFs (Figure 4A–E), whereas decreased by Ad-siRUNX1 in C2C12 and MEFs (Figure 4F–J). These results indicate that RUNX1 can regulate BMP9-induced mRNA expression levels of pivotal osteogenic markers in MSCs and MMCs.

### 2.6. Effect of RUNX1 on BMP9-Induced Protein Expression Levels of Pivotal Osteogenic Markers

**Figure 5:**
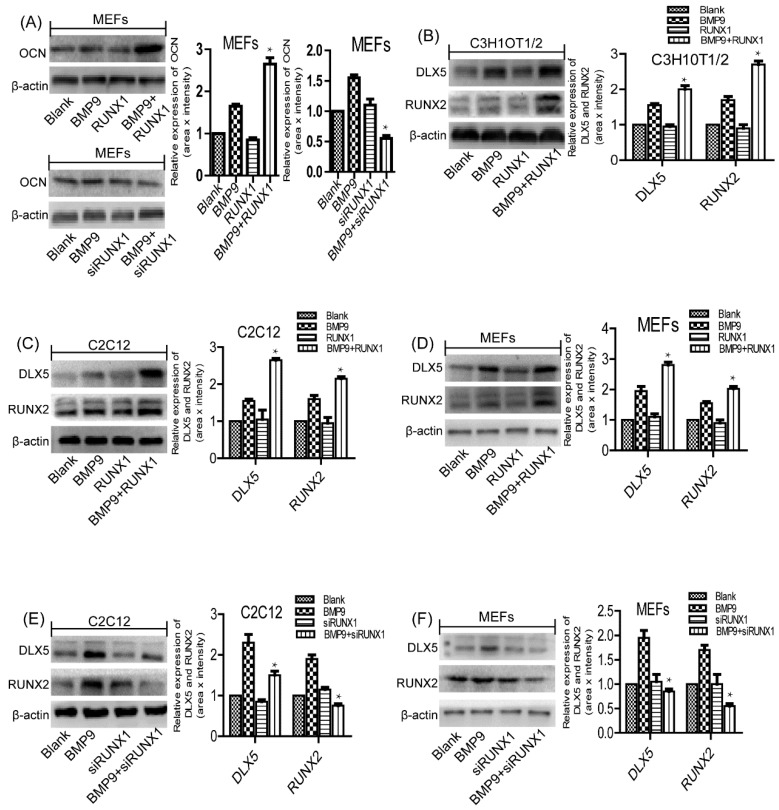
Effect of RUNX1 on BMP9-induced protein expression levels of pivotal osteogenic markers. Cells were infected with Ad-RUNX1 or Ad-siRUNX1, and then treated by optimal BMP9-CM. Total protein was extracted and was measured by Western blot. (**A**) Overexpression of RUNX1 promoted the expression of BMP9 induced OCN in protein levels, while its deficiency using Ad-RUNX1 reduced OCN expression. Overexpression of RUNX1 promoted BMP9-induced protein expression levels of DLX5 and RUNX2 in: C3H10T1/2 (**B**); C2C12 (**C**); and MEFs (**D**). Inhibition of RUNX1 decreased BMP9-induced protein expression of DLX5 and RUNX2 in: C2C12 (**E**); and MEFs (**F**). * *p* < 0.05, as compared with BMP9 group.

As the relationship between mRNA and protein levels is not always straightforward, to further clarify the role of RUNX1 in BMP9 osteoinductive activity, we employed Western blot to analyze the effect of RUNX1 on BMP9-induced protein expression levels of pivotal osteogenic markers. Consistent with the mRNA level, the protein expression of BMP9-induced OCN was significantly increased by Ad-RUNX1 and decreased by Ad-siRUNX1 in MEFs (Figure 5A). Subsequently, we further examined the protein expression levels of DLX5 and RUNX2, and found that Ad-RUNX1 could enhance BMP9-induced protein expression of DLX5 and RUNX2 in C3H10T1/2 (Figure 5B), C2C12 (Figure 5C) and MEFs (Figure 5D), while Ad-siRUNX1 could decrease the protein expression of DLX5 and RUNX2 in C2C12 (Figure 5E) and MEFs (Figure 5F). Collectively, these above results suggest that RUNX1 is involved in regulating BMP9-induced osteogenic differentiation of MSCs and MMCs.

### 2.7. Effect of RUNX1 on BMP9-Induced Classical Smad1/5/8 Signaling and MAPKs Signaling

**Figure 6:**
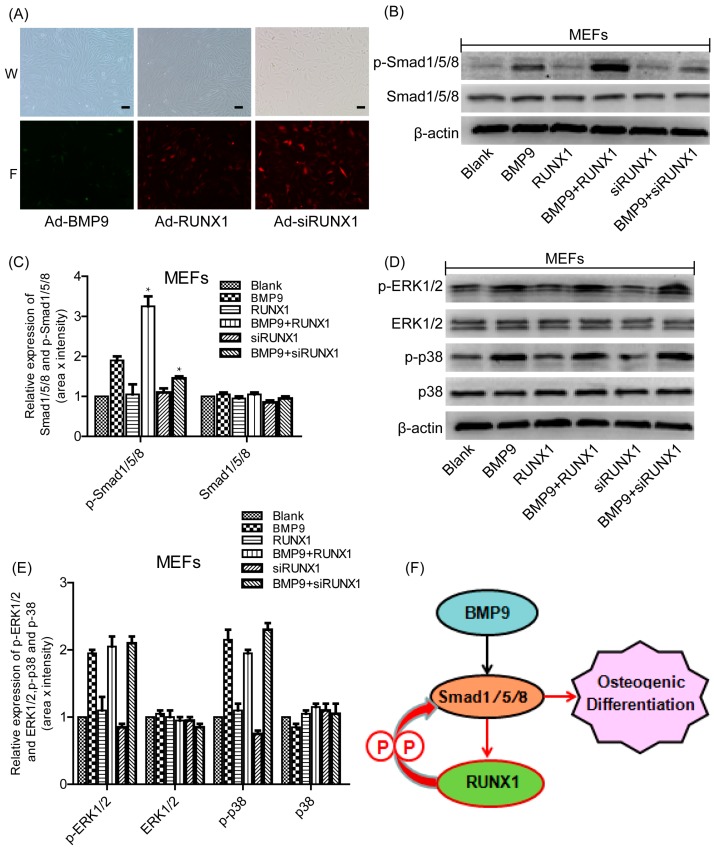
Effect of RUNX1 on BMP9-induced classical Smad1/5/8 signaling and MAPKs (Mitogen-activated protein kinases) signaling. Cells were infected with Ad-RUNX1, Ad-siRUNX1, and Ad-BMP9, respectively. Total protein was extracted and was measured by Western blot. (**A**) Ad-BMP9, Ad-RUNX1 and Ad-siRUNX1 could infect MEFs cell; W (white light), F (fluorescence). (**B**,**C**) Effect of RUNX1 on BMP9-activated Smad1/5/8 signaling. (**D**,**E**) Effect of RUNX1 on BMP9-induced p38 and ERK1/2 (Extracellular signal-regulated protein kinase1/2) signaling. (**F**) A schematic presentation of proposed mechanism of BMP9/RUNX1 regulation of osteogenesis, P (phosphorylation), black arrow (Other studies confirm the phenomenon), red arrow and curved red arrow (our study confirm the phenomenon). * *p* < 0.05, as compared with BMP9 group. Scale bar represents 50 μm Magnification, ×100.

We then sought to investigate the possible mechanisms responsible for the effects of RUNX1on BMP9-induced osteogenic differentiation. Studies have reported that BMPs bind to the extracellular domain of the BMP receptors (serine/threonine kinase receptors; types I and II) and these in turn activate the canonical Smad-dependent pathway and Smad-independent pathway that are responsible for modulating gene transcription[13,14]. Therefore, we tried to explore whether BMP9-activated Smads signaling and MAPKs signaling were influenced by RUNX1. We infected MEFs with Ad-RUNX1, Ad-siRUNX1 and Ad-BMP9 (Figure 6A) and found that Ad-RUNX1 promoted BMP9-induced phosphorylation of Smad1/5/8, whereas Ad-siRUNX1 inhibited BMP9-induced phosphorylation of Smad1/5/8 (Figure 6B,C). However, The BMP9-induced phosphorylation of p38 and ERK1/2 was not altered by treatment with Ad-RUNX1 or Ad-siRUNX1 (Figure 6D,E). Taken together, these results demonstrate that RUNX1 regulates BMP9-induced differentiation of MSCs and MMCs via canonical Smad signaling.

## 3. Discussion

BMP9 (also known as growth differentiation factor 2 (GDF-2)) was originally identified in the developing mouse liver, and has an important role in iron metabolism and the development of cholinergic neurons [38,39]. Other roles of BMP9 includes stimulating of hepatocyte proliferation and regulating glucose and lipid metabolism in liver [40]. BMP9 is also known to exert several biological effects on tumor cells. It has been reported that BMP9 can promote the growth of ovarian cancer cells [41], whereas inhibit the growth and invasion of prostate cancer PC-3 cells through the BMP9-induced classical Smad-dependent signaling and Smad-independent MAPKs signaling [42]. Ren et al found that BMP9 also has the effect on promoting the apoptosis of breast cancer cells and inhibit their invasion and migration capacities via the down regulation of CTGF [43]. Although BMP9 has been previously identified as one of the most robust osteogenic BMPs both in vitro and in vivo [44], the detail mechanism remains unclear.

Mammalian runt-related transcription factor RUNX family members including RUNX1, RUNX2 and RUNX3 are closely involved in embryonic development [45]. Our team has discovered that RUNX2 was essential for BMP9-induced osteogenesis [46]. Moreover, RUNX family members share a similar structure and co-distributed in skeletal elements, and RUNX1 is an important regulator during chondrocyte differentiation and bone formation [47,48,49]. Based on these above research results, we proposed that RUNX1 might be also involved in BMP9-induced osteogenic differentiation. In this current study, we found that BMP9 can promote the expression of RUNX1 in MSCs and MMCs. ChIP analysis revealed that RUNX1 was probably a direct target of the BMP9-specific Smad1/5/8. Besides, we also confirmed that overexpression of RUNX1 can promote BMP9-induced osteogenic differentiation and knockdown of RUNX1 had the opposite effect. Smad1/5/8 signaling and MAPKs signaling are the most important pathways involved in regulating BMP9-induced osteogenic differentiation [50]. We demonstrated that the phosphorylation of Smad1/5/8 was increased by overexpression of RUNX1 and decreased by knocking down of RUNX1. One possible explanation of the RUNX1 effect on Smad1/5/8 signaling is that RUNX1 may facilitate Smad1/5/8 to bind to BMP type I receptor which can directly phosphorylate Smad1/5/8. Additionally, a variety of molecules are involved in the regulation of BMP-specific Smad1/5/8 signaling, such as SARA (Smad anchor for receptor activation) [51], nuclear export factor RanBP3 [52], and Nuclear phosphatase PPM1A [53]. Thus, another explanation of the RUNX1 effect on Smad1/5/8signaling is that RUNX1 may interact with these molecules t to affect Smad1/5/8 phosphorylation. Although BMP9 can simultaneously stimulate phosphorylation/activation of p38 and ERK1/2 [24], it seems that RUNX1 did not affect the BMP9-induced phosphorylation of p38 and ERK1/2. Together, our results strongly indicate that RUNX1is likely to play a regulatory role in BMP9-induced osteogenic differentiation of MSCs and MMCs mainly through affecting Smad1/5/8 signaling.

Previous studies have demonstrated that RUNX1 was a significant regulator in chondrocyte differentiation [30,32]. To date, little is known about RUNX1 during osteogenesis. In this study, we investigated the detailed roles of RUNX1 during BMP9-induced osteogenic differentiation. We demonstrated that BMP9 can promote the expression of RUNX1 through Smad1/5/8 signaling, and RUNX1 may regulate osteogenic differentiation of MSCs and MMCs by affecting BMP9-induced Smad1/5/8 phosphorylation/activation (Figure 6F). As one member of RUNX family, RUNX2 is a pivotal osteogenic transcription factor, and can induce osteoblast differentiation in both endochondral and intramembranous ossification [31]. We have demonstrated that RUNX2 promoted BMP9-induced osteogenic differentiation of MSCs, this finding was consistent with the conclusion that RUNX2 is one of the downstream target genes of BMPs family [28]. The role of RUNX1 in BMPs family was unclear by now. RUNX1 was expressed not only by cartilage precursor cells and pre-osteoblast, but also by early mesenchymal stem cells [30]. In these cells, overexpression of RUNX1 promoted chondrocyte differentiation and knockdown RUNX1 inhibited RUNX2 expression as well as chondrocyte and osteoblast differentiation [30]. In our study, we validated that RUNX1 affected BMP9-induced expression of RUNX2.These results suggested that RUNX1 might bean upstream molecule of RUNX2. Moreover, we also found that RUNX1 regulated BMP9-induced expression of OCN. However, our previous study showed that RUNX2 did not affected BMP9-induced expression of OCN [28,34].These results suggested that RUNX1 and RUNX2 may have different mechanisms of action in BMP9-induced osteogenic differentiation of MSCs. Totally, our findings in this current study may contribute to enrich understanding about osteoinductive activity of BMP9.Future studies should be devoted to characterize the direct targets of RUNX1 during BMP9-induced osteogenesis, investigate the precise mechanism of RUNX1 in affecting phosphorylation of Smad1/5/8 and verify these results in an in vivo system.

## 4. Materials and Methods

### 4.1. Cell Lines and Chemicals

HCT116 (colorectal carcinoma), MEFs (murine embryonic fibroblast), C2C12 (murine myoblasts) and C3H10T1/2, (obtained from the American Type Culture Collection, ATCC). Dulbecco’s modified Eagle’s medium (DMEM) and fetal bovine serum (FBS) (both from Gibco, Grand Island, NY, USA). Anti-RUNX1,anti-RUNX2,anti-phospho-Smad1/5/8,anti-ERK1/2, anti-phospho-ERK1/2, anti-p38, and anti-phospho-p38 antibodies (purchased from Cell Signaling Technology, Danvers, MA, USA), Anti-Smad1/5/8 (sc-6031), anti-osteocalcin, anti-distal-less homeobox 5 and anti-β-actin (obtained from Santa Cruz Biotechnology, Inc., Santa Cruz, CA, USA) were used. Unless indicated, all chemicals were purchased from Sigma-Aldrich (Saint Louis, MO, USA).

### 4.2. Construction of Recombinant Adenoviruses

Recombinant adenovirus expressing exogenous BMP9 (Ad-BMP9) and RUNX1 (Ad-RUNX1), and recombinant adenovirus expressing small interfering RNA (siRNA) targeted RUNX1 (Ad-siRUNX1) were generated previously using the AdEasysystem, as demonstrated [54]. Adenoviruses only expressing GFP (Ad-GFP) and RFP (Ad-RFP) were used as controls, which were kindly provided by Tong-chuan He of University of Chicago Medical Center(Chicago, IL, USA).

### 4.3. Cell Culture

MSCs and other cell lines were cultured in Dulbecco’s modified Eagle’s medium (DMEM; Hyclone, South Logan, UT, USA) supplemented with 10% fetal bovine serum (FBS; Gibco, Junction City, KS, USA), 1% penicillin, and 1% streptomycin. All cells were cultured at 37 °C in a 5% CO_2_ incubator under humidified atmosphere.

### 4.4. Preparation of Conditioned Medium

BMP9 conditioned media (BMP9-CM) were prepared as described [55].Briefly, HCT116 cells were seeded in 100 mm dish and infected with an optimal titer of Ad-BMP9. At 4 h after infection, the culture medium was changed to serum free DMEM. Conditioned medium (BMP9-CM) was harvested at 24 h and 48 h after infection and used immediately.

### 4.5. Chromatin Immunoprecipitation (ChIP) Analysis

Ad-GFP or Ad-BMP9 infected subconfluent MEFs cells. At 48 hours after infection, cells were cross-linked and subjected to ChIP analysis as previously described [56,57,58]. Smad1/5/8 antibody (Santa Cruz Biotechnology) or control IgG was used to pull down the protein-DNA complexes. The presence of RUNX1promoter sequence was detected by using two pairs of primers corresponding to mouse RUNX1 promoter region.

### 4.6. Alkaline Phosphatase (ALP) Assays

ALP activity was assessed by a modified Great Escape SEAP Chemiluminescence assay (BD Clontech, Mountain View, CA, USA) and histochemical staining assay (using a mixture of 0.1 mg/mL of napthol AS-MX phosphate and 0.6 mg/mL of Fast Blue BB salt) as described previously [27]. Each assay condition was performed in triplicate and the results were repeated in at least three independent experiments. ALP activity was normalized by total cellular protein concentrations among the samples.

### 4.7. Measurement of Matrix Mineralization

Mineralized matrix nodules were stained for calcium precipitation by means of Alizarin Red S staining, as described previously [59]. Cultured cells were seeded in 24-well cell culture plates, and were cultured in the presence of ascorbic acid (50 mg/mL) and β-glycerophosphate (10 mM) after being infected with Ad-RUNX1 or Ad-siRUNX1followed by treated with BMP9-CM. At 14 days after cultured, mineralized matrix nodules were stained for calcium precipitation by means of Alizarin Red S staining.

### 4.8. Total RNA Isolation, Semi-Quantitative PCR (RT-PCR) and Quantitative Real-Time PCR (RT-qPCR)

Total RNA was isolated using TRIzol reagent (Invitrogen, Carlsbad, CA, USA) according to the RNA extraction protocol. Total RNA was used for cDNA synthesis by reverse transcriptase PCR. GAPDH was used as the endogenous control. PCR was carried out as described previously [60]; PCR primers (Table 1) were designed using Primer3 program. A touchdown cycling program for RT-PCR was carried out as follows: 94 °C for 5 min for 1 cycle, 94 °C for 30 s, 68 °C for 30 s, and 72 °C for 12 cycles with decrease in 1 °C /cycle and then at 94 °C for 30 s, 55 °C for 30 s, and 72 °C for 30 s for 18–27 cycles depending on the abundance of a given gene. The cDNA was amplified by a real-time polymerase chain reaction (RT-qPCR) system (Bio-Rad, USA) using SYBR Green PCR Master Mix. Gene expression results were analyzed using the ΔΔ*C*_t_ method. Reaction conditions were as follows: 95 °C for 3 min, 95 °C for 3 s, 55 °C for 30 s (36 cycles), 95 °C for 10 s, 65 °C for 10 s and 95 °C for 50 s.

### 4.9. Western Blot

The cells were washed three times with cold PBS and lysed in RIPA lysis buffer (Beyotime, Haimen, China), Cell lysis solution was centrifuged at 13,000× *g* for 15 min at 4 °C and protein solution were collected. The BCA (Bicinchoninic acid) protein assay kit (Beyotime, Haimen, China) was used to measure the protein concentration. Equivalent amounts of protein were loaded in SDS-PAGE (sodium dodecyl sulfate polyacrylamide gel electrophoresis) polyacrylamide gels and then transferred onto PVDF (polyvinylidene fluoride) membranes. The PVDF membranes were blocked with 5% bovine serum albumin (BSA; Solarbio, Beijing, China) in TBST for 2h at 37 °C. Subsequently, the membranes were incubated with the primary antibodies overnight at 4 °C. Then, the membranes were washed with TBST 3 times and incubation with a secondary antibody (1:5000, ZhongShan-Golden Bridge, Beijing, China) for 1 h at 37 °C. The Super Signal West Pico Chemiluminescent Substrate kit was used to quantify protein levels, as described previously [61].

## Figures and Tables

**Table 1:** Sequences of primers for RT-PCR (Semi-Quantitative PCR) and RT-qPCR (Quantitative Real-Time PCR).

Gene Name	Forward Primer	Reverse Primer
*GAPDH*	GGCTGCCCAGAACATCAT	CGGACACATTGGGGGTAG
*CollaI*	ATCGACATGTCAGCCTTTGC	TGACTTGAGTGTAGCGTCCAC
*OSX*	GGGAGCAGAGTGCCAAGA	TACTCCTGGCGCATAGGG
*DLX5*	CTCAGCCACCACCCTCAT	TGGCAGGTGGGAATTGAT
*RUNX2*	GGTGAAACTCTTGCCTCGTC	AGTCCCAACTTCCTGTGCT
*RUNX1*	GCCATGGCTACGGTTCAG	CAGAACCAGCGGTTAGGC
